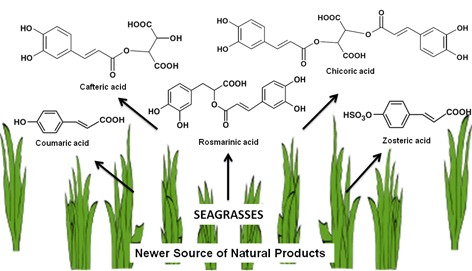# Bioactive natural products from marine angiosperms: abundance and functions

**DOI:** 10.1007/s13659-013-0043-6

**Published:** 2013-08-05

**Authors:** Ponnambalam Subhashini, Elangovan Dilipan, Thirunavukkarasu Thangaradjou, Jutta Papenbrock

**Affiliations:** 1 Centre of Advanced Study in Marine Biology, Annamalai University, Parangipettai, 608502 Cuddalore Dt., Tamil nadu India; 2 Institute of Botany, Leibniz University Hannover, Herrenhäuserstr. 2, D-30419 Hannover, Germany

**Keywords:** natural products, phenolic compounds, phenylpropanoid derivatives, seagrasses, secondary metabolites

## Abstract

This review explores the natural products of seagrass that are to be exploited for their bioactive potential. Beside from portraying the presence of a wide array of secondary compounds such as phenols, flavonoids, sterols and lipids from different seagrass species, the focus is on novel natural products projecting towards their biological applications. Though there are a significant number of reports on the abundance of secondary metabolites from seagrass and their bioactive derivatives, only a small number of reports explore their functional and defensive characteristics. Efforts have been made to collate the available information on seagrass natural products and clarify their function and metabolic pathway’s. It is emphasized that metabolic profiling of seagrass should be extensively progressed to obtain a deeper knowledge about the specific roles of each natural product. The investigation of seagrass natural products for their bioactive potential would most likely result in the detection of surprising and unexpected novel chemical structures and clinical leads that may be useful to mankind.

## 1 Seagrasses—the Marine Angiosperms

Seagrasses, a functional group of flowering plants rooted in the world's coastal oceans, are well known for their secondary metabolites. The current comprehensive review on "Bioactive natural products from marine angiosperms'' was performed in such a way that recent discoveries in the field of seagrass secondary metabolism need to be reviewed. In this review is not possible to detail in a just few pages the full gamut of studies, since our understanding of natural products from seagrasses has increased markedly in the last few decades. Several studies have documented the abundance of these natural metabolites in seagrasses and some have focused on their bioactive potential. Though there are probably 72 species of seagrasses worldwide, only a few have been explored for their natural products and their determinant role. Recently, the secondary metabolites of *P. oceanica* were compiled[1], appraised and were summarized into 51 natural products including phenols, phenylmethane, phenylethane, phenylpropane derivatives and their esters, chalkones and flavonoids. These chemical compounds synthesized by secondary metabolic pathways are not involved in the normal growth, development or reproduction but usually have roles in adaptation processes under stress conditions.

A severe or long lasting stress factor could induce an excessive shift between primary and secondary metabolism and consequently, a diversion of essential available resources from growth to defense[2], since the products of primary metabolism are the precursors for secondary metabolic pathways. The topics provided in this paper are limited to seagrass secondary metabolism although it has become clear that a clear line between primary and secondary metabolism cannot be drawn. Hence, the present review aims to collate the information available on the natural products of seagrasses, their biogenesis, the metabolic sequence leading to their formation and their biological functions in general, and, in seagrass in particular.

## 2 Biogenesis of Natural Products

An overview of seagrass metabolism, shows that the basic pool of secondary metabolites is similar to their terrestrial relatives from which they have evolved[3]. The biosynthesis of most secondary metabolites begins with a relatively small group of compounds, which are modified into an unlimited number of compounds through various synthesis pathways. The most important of these metabolic pathways are those with shikimic acid, acetyl coenzyme A (acetyl-CoA), and mevalonic acid intermediates, known as the shikimate, acetate

*To whom correspondence should be addressed. E-mail: umaradjou@gmail.com

**Figure 1.** Schematic overview of the biosynthetic routes involved in the natural product synthesis and polyketide pathways, respectively (Figure 1). Of the three pathways, the shikimate pathway is the major defense pathway in plants by which the phenylpropanoid and flavonoid derivatives are synthesized forming the bulk of metabolites[4]. The biogenesis of natural products derived from these three pathways in seagrasses is described in detail below.

### 3 Derivatives of Shikimate Pathway

Being the key intermediate from a combination of phosphoenolpyruvate (a glycolytic pathway intermediate) and erythrose-4-phosphate from the pentose phosphate pathway for the biosynthesis of $C_6$-$C_3$ units (phenyl propane derivatives), shikimic acid acts as a precursor for the biosynthesis of amino acids. It also triggers the production of metabolites such as tannin, vanillin, lignin, cinnamic acid derivatives and flavonoids along with simple phenol derivatives. Also, shikimic acid leads to the synthesis of aromatic amino acids, aliphatic amino acids and finally to peptide alkaloids. Of all such compounds, the phytochemistry of seagrasses put forth by different scientists from all over the world has revealed the presence of various excess phenylpropanoid derivatives.

**3.1 Phenylpropanoids:** Phenylpropanoids include simple phenol, phenylmethane, phenylethane derivatives and lignin precursors. Phenolic compounds, such as simple phenol derivatives are common constituents of vascular plants and may be separated into three operationally-defined classes: soluble, ester-bound and lignin-derived[5]. These compounds appear to have a myriad of supplemental functions in a plant's life cycle such as structural roles in different supporting or protective tissues, involvement in defense strategies, as attractants for pollinators and seed-dispersing animals, and as allelopathic agents, ultraviolet protectants and signal molecules in the interactions between plants and their environment[6].

**3.2 Simple Phenolic Compounds:** Seagrasses are a rich source of phenolic substances including phenolic acids, sulphated phenolic acids, flavones, condensed tannins and lignins, but not hydrolyzable tannins[7,8]. Ester-bound phenols may be linked to lignin[9] or cell-wall carbohydrate[10] and are degraded at considerably lower rates than soluble phenols[5]. Soluble polyphenolic contents of tropical seagrasses were measured quantitatively[11,12], and also using fourier transform infrared spectroscopy[13]. The phenolic acids such as *p*-coumaric acid, caffeic acid, ferulic acid, protocatechuic acid, *p*-hydroxybenzoic acid, vanillic acid, gentisic acid and gallic acid are found in terrestrial plants and occur predominantly in *Halophila ovalis*, *Thalassia hemprichii*, *Halodule* spp., *Cymodocea* spp., *Enhalus acoroides*, *Syringodium isoetifolium* and other seagrass species[14,15,16]. These simple phenolic compounds are mostly hydroxybenzoic and hydroxycinnamic acid derivatives (Figure 2). The array of phenolic acids

**Figure 2.** (A) Hydroxybenzoic acids (B) Hydroxycinnamic acids produced by seagrasses is dependent upon the species, population, and tissue examined.

About 53 polyphenolic compounds were identified from the above-ground samples of *P. oceancia* and *Z. marina* using GC-MS[17]. Similarly, with the predominance of caffeic acid in the adult and intermediate leaves of *P. oceanica*, other phenolic compounds such as 4-hydroxybenzoic acid, 4-coumaric acid, trans-cinnamic acid, caffeic acid and a mixture of ferulic acid and ester methyl 12-acetoxyricinoleate were also reported[18]. The difference existing in both the free and bound phenolics of *P. oceanica* were estimated in samples collected from different geographic regions[19,20]. Similarly, phenol content is found to vary in different parts of rhizomes in *P. oceanica*[21]. The simple phenols and their derivatives from different seagrasses are presented in Table 1. Amongst all the seagrass species of the world, the temperate species *P. oceanica* has been most extensively studied for its natural product composition followed by *Z. marina*, when compared with other seagrasses that makes it difficult to tabulate them completely for comparative purposes. From the existing literature, it is confirmed that the techniques, age of the plant, part of the samples, extraction procedure are all playing crucial roles in determining the phenol content.

**3.3 Phenylmethane and Phenylethane Derivatives:** Seagrasses also accumulate significant levels of condensed tannins (proanthocyanins) in leaf and shoot tissues. Lignin contents vary as well, from < 1 to 5% dry mass, but remain slightly lower than concentrations found in terrestrial grasses[22]. Phenylmethane derivatives like *p*-hydroxybenzoic acid and vanillic acid are abundant in the roots and vanillic acid in the leaves of *P. oceanica*[23] and also esters of phenylpropanic acid derivatives of (2*R*,3*R*)-cichoric acid have been identified from the leaves of the same species. Simple phenol derivatives, phenylmethane, phenylethane and phenylpropane derivatives have been identified in the leaves of *P. oceanica*[24] and a total of 23 phenolic compounds from *P. oceanica* collected at different geographic regions[25] illustrate seagrasses as a promising source of polyphenolic compounds. Phenylmethane and phenylpropane derivatives from the leaves of *P. oceanica* were detected by comparing HPLC retention time and co-chromatography with authentic reference compounds[26].

The detrital and fresh leaves of *S. filiforme*[27] and fresh leaves of *P. oceanica*[26] are enriched with chicoric acid and cafteric acid. Rosmarinic acid (RA), the caffeoyl ester, has a broad range of applications from food preservatives to cosmetics with high antioxidant activity. This therapeutically potent polyphenolic compound that has been isolated from *Zostera marina*[16], that confirms seagrasses to be a new source of RA. In contrast, caffeic acid which is the precursor for the formation of RA in the shikimic acid biosynthesis, was found only in trace amounts. Similarly, the occurrence of RA with other phenols like caffeic acid and chlorogenic acid were traced from the leaves of *Z. noltii*[28]. Similarly, zosteric acid (ZA), chemically *p*-(sulfooxy) cinnamic acid, becomes noteworthy for its anti-fouling activity[29], which has been proved to inhibit the growth of biofilm forming bacteria[30,31] and fungal spores[32]. Both the living leaves[33] and the detritus of the seagrasses *Z. noltii* and *Z. marina* serve as a rich source of ZA that could justify significant exploitation of this low cost, very abundant and renewable, but relatively unused, marine resource[34]. Chemical structures of the phenolic compounds from seagrasses are presented in Figure 3.

**3.4 Flavonoid Derivatives:** Flavonoids, a class of polyphenolic compounds, occur in any of the five chemical structures like flavones, flavonols, flavanons, flavanols and anthocyanidins. The presence of sulfated flavones was indicated in *Halophila*, *Thalassia* and *Zostera* species[35], but they were not recorded in *Syringodium* spp. or *P. oceancia*. Flavonoid sulfates were also traced out in *Halophila ovalis* and *Thalassia testudinum*[36,37]. Forty three species of seagrasses were exclusively studied and identified that all contained either flavones and/or phenolic acid sulfates[36]. Among the 12 genera examined, five (*Zostera*, *Phyllospadix*, *Enhalus*, *Thalassia* and *Halophila*) had sulfated flavones. The occurrence of the sulfated phenolic compounds indicated subgeneric differences in *Zostera* and interspecific differences in *Halophila*. Similarly, sulfated flavonoids were reported from the seagrass *Z. marina*[38]. The lyophilized aqueous extract of *T. testudinum* yielded three sulfated flavones (Figure 4), thalassiolin A (luteolin-7-O-*β*-D-glucopyranosyl-2-sulfate), thalassiolin B (3-methyoxyluteolin-7-*β*-D-glucopyranosyl-2-

**Table 1. Simple phenol and phenylpropanoid derivatives from seagrasses**

| Seagrass | 1 | 2 | 3 | 4 | 5 | 6 | 7 | 8 | 9 | 10 | 11 | 12 | 13 | 14 | 15 | 16 | 17 | 18 | 19 | 20 | References |
|---|---|---|---|---|---|---|---|---|---|---|---|---|---|---|---|---|---|---|---|---|---|
| *Halodule spp.* | + | + | – | – | + | – | + | – | – | – | – | – | – | – | – | – | – | – | – | – | 11, 15 |
| *Halophila ovalis* | + | + | + | + | + | + | – | + | – | – | – | – | – | – | – | – | – | – | – | – | 11, 15 |
| *Thalassia hemprichii* | + | + | + | + | + | + | – | – | – | – | – | – | – | – | – | – | – | – | – | – | 11, 15 |
| *Cymodocea* spp. | + | + | – | – | + | + | + | – | – | – | – | – | – | – | – | – | – | – | – | – | 11, 15 |
| *Enhalus acoroides* | + | – | + | + | + | + | – | – | – | – | – | – | – | – | – | – | – | – | – | – | 11, 15 |
| *Syringodium isoetifolium* | – | + | + | + | + | + | + | + | – | – | – | – | – | – | – | – | – | – | – | – | 11, 15 |
| *Zostera marina* | + | + | + | – | – | + | – | + | + | + | – | + | + | + | + | + | + | + | + | + | 16, 17, 77, 34 |
| *Zostera noltii* | – | – | – | – | – | – | – | – | – | + | – | – | – | – | – | – | – | – | – | – | 81 |
| *Posidonia oceanica* | + | + | + | + | + | + | + | + | + | – | + | – | + | + | + | + | + | + | + | + | 17, 82 |
| *Syringodium filiforme* | – | – | – | – | – | – | – | – | – | – | + | + | – | – | – | – | – | – | – | – | 27 |

1 *p*-Coumaric acid, 2 Caffeic acid, 3 Ferulic acid, 4 Protocatechuic acid, 5 *p*-Hydroxy benzoic acid, 6 Gallic acid, 7 Vanillic acid, 8 Phenol, 9 Benzoic acid, 10 Rosmarinic acid, 11 Chicoric acid, 12 Cafteric acid, 13 *O*-Methylphenol, 14 *p*-Methylphenol, 15 Ethylphenol, 16 Guaiacol, 17 Vinylphenol, 18 Hydroxyguaiacol, 19 Vinylguaiacol, 20 Zosteric acid

**Figure 3.** Chemical structures of phenolic compounds from seagrasses sulfate) and thalassiolin C (apigenin-7-$\beta$-D-glucopyranosyl-2-sulfate)[37].

Other flavonoid derivatives gaining attention are those from the seagrass *Phyllospadix japonica.* Flavonoids such as luteolin-5-methylether, luteolin-5,4-dimethylene, acacetin-5-methyl ether, pectolinarigenin, hispidulin, jaceosidin, 5,7,3-trihydroxy-6,4-dimethoxyflavone and 6-hydroxyluteolin[39], and three other flavone acetates namely luteolin acetate, hispidulin acetate and 5,7,4′-trihydroxy-6-methoxy-8-(1-methyl-2-pyrrolidinyl) and a flavone[40] were isolated. Interestingly the flavone isolated was a flavonoidal alkaloid, is the only alkaloid derivative ever isolated from a seagrass species. *Zostera* spp. has yielded with good fractions of flavonoids like luteolin-7-sulphate and luteolin-7,3′-disulphate (*Z. marina*)[41], luteolin-7-O-glucoside, diosmetin and diosmetin-7-O-glucoside (*Z. marina* and *Z. nana*)[42], and luteolin-7-O-glucoside (*Z. noltii*)[28]. These functional derivatives of flavonoids are considered to defend against the marine microorganisms by exhibiting a chemical defense.

Flavonoid glycosides and acyl derivatives, yield the respective flavonoid aglycones after hydrolysis in *P. oceanica* leaves[43]. Four flavones namely, luteolin, apigenin, luteolin-3-glucoronide and luteolin-4-O-glucoronide were identified from the ethanol extract of air dried *Enhalus acoroides* from South China Sea[44]. Seven new flavonoid glycoside along with five flavones, and three flavonoid glycosides from *Halophila johnsonii*[45] and malonylated flavone glycoside derivatives from *Halophila stipulaceae*[46] suggest that seagrasses are comparatively stronger in natural product synthesis in their stressful environment. Significantly higher flavonoid amounts were observed in the leaves of intertidal and subtidal *H. johnsonii* when compared to the leaves of intertidal *H. decipiens*[47]. Major flavonoid compounds identified from seagrasses are presented in Figure 5 and the intermediate metabolites from the shikimate pathway with other few metabolites reported from seagrasses are listed in Table 2.

## 4 Derivatives of Acetyl-Co A Pathway

**4.1 Sterols and Volatile Derivatives:** The sterol composition of temperate seagrasses is found to be widely explored when compared to the tropical seagrasses. The sterols of *P. oceanica* and *Cymodocea nodosa* are essentially $\beta$-sitosterol and sigmasterol, with minor sterols including cholesterol, campesterol and avenasterol[48,49]. The component sterols, alcohols, hydrocarbons, monocarboxylic, $\alpha,\omega$-dicarboxylic and $\alpha$- and $\omega$-hydroxy fatty acids were identified from the leaves and roots of the tropical seagrass *T. hemprichii*[50]. Similarly, $\alpha$-hydroxy fatty acids have been reported in the leaves of *Z. mulleri* and in associated sediments[51].

Forty four volatile compounds were reported from *Z. marina* species using GC-MS analysis[52], in which they found

**Figure 4.** Thalassiolins identified from the seagrass *T. testudinum*

that major constituents were phytol, hexadecanamide, octadecanamide, pentadecane, heptadecane, nonadecane, (8*Z*,11*Z*)-heptadecadienal (HDD), (8*Z*)-heptadecenal (HD), (9*Z*,12*Z*,15*Z*)-octadecatrienal and (9*Z*,12*Z*)-octadecadienal. The vast data base available for the secondary metabolites of the *Zostera* spp. and *P. oceanica* has to be analyzed in detail to find identifying marker compounds. The presence of polyphenols in most of the seagrasses also helps to preserve the fatty acids as polyphenols are known for their ability to prevent fatty acids from oxidative decay, and that provide a defense against the oxidative stress of oxidizing agents and free radicals[53].

**Table 2. Different metabolites reported from seagrasses**

| Seagrass species | Metabolite | Reference |
|---|---|---|
| *Zostera marina* | Sulfated flavones | 35 |
| *Halophila stipulacea* | Malonyl derivatives | 46 |
| *Halophila johnsonii* | Flavone glycosides | 45 |
| *Thalassia testudinum* | Flavone glycoside | 65 |
| *Halophila ovalis*, *H. minor* | Sulfated flavonoids | 83 |
| *Thalassia testudinum* | Condensed tannins | 22 |
| *Thalassia testudinum* | Thalassiolins | 37 |
| *Syringodium filiforme* | L-chiro-Inositol | 84 |
| *Cymodocea nodosa* | Diarylheptanoids | 68 |

**4.2 Lipids and Fatty Acids:** The Fatty acids from lipids of *P. oceanica* collected in France and Greece were analyzed in detail using GC/FID in comparison with retention times of reference compounds[54] that portrayed long chain fatty acids (C22-C34) as a feature setting *P. oceanica* apart from sympatric species of algae, thus possibly enabling identification of *P. oceanica* residues in marine sediments. This can be effectively used in biogeochemical studies. However, the presence of such long chain fatty acid should also be confirmed in other seagrass species. Polar lipid composition and fatty acids from *Z. marina* and *Phyllospadix iwatensis*[55] and major phospholipids (phosphatidylcholine and phosphatidylethanolamine) and glycolipids (monogalactosyldiacyl glycerol and digalactosyldiacylglycerol) have been reported from *Z. marina*[56]. Sequentially, the fatty acid composition of phospholipids, glycoglycerolipids and betaine lipids of *Z. marina* undergoes seasonal changes with physico-chemical parameters so as to adapt with the environment. Analysis of lipids revealed a common increase of the ratio between n-3 and n-6 polyunsaturated fatty acids (PUFAs) of polar lipids from summer to winter despite their chemotaxonomically different fatty acid composition[57,58].

## 5 Functions of Seagrass Natural Products

The concentration of phenolic compound was measured in the seagrass *P. oceanica* when interacting with *Caulerpa taxifolia* and *Caulerpa racemosa*. Five phenolic compounds were identified in *P. oceanica*, with a predominance of caffeic acid in the adult and intermediate leaves. The number of tannin cells (supposed to produce the phenolic compounds) largely increased in the adult and intermediate leaves when the degree of interaction with *C. taxifolia* increased. It thus appears that when the seagrass *P. oceanica* interacts with *C. taxifolia*, it accelerates its production of secondary metabolites to limit invasion of the beds[18]. This also suggests the presence of phenols as defense mechanisms in seagrass prevent the micro- and macroalgal epiphytes.

**5.1 Chemotaxonomic Tool:** Systematic profiling of the major and specific secondary metabolites for several seagrasses can be collated and used as a chemotaxonomic tool for identification. The nature of different biotic and abiotic interactions in which phytochemicals participate warrants a high degree of variability in their distribution, even amongst related plants limiting their value as taxonomic tools[59]. The presence of flavanoid derivatives in *H. stipulacea* has been revealed[46] which establishes them as important information for relating flavones as a typical taxonomic tool particularly for the genus *Halophila*. It is proposed that high proportion of cholest-5-en-$\beta$-ol detected in *T. hemprichii* can be used as a

**Figure 5.** Chemical structure of the flavonoid compounds from seagrasses chemotaxonomic feature of this seagrass[50]. From the literature, it is evident that sulfated phenols have the key to demarcate seagrasses at least to generic level. Studies have portrayed that *Heterozostera* lacks sulfated flavones[36] which are characteristic of *Zostera* and *Phyllospadix* of Zosteraceae, and also in all the other species of Posidoniaceae and Cymodoceaceae. The major phospholipid composition of seagrasses, phosphatidylcholine (41.8–43.8%) and phosphatidylethanolamine (22.4–24.3%) and N-acylphosphatidylethanolamine that differs from that of seaweeds, can be used as a taxonomic tool[60]. Detailed screening of other seagrasses could able to identify the universal or species specific markers which can be used for identification of species and also as biogeochemical marker. Phytochemical variation of the three genetically defined groups found by microsatellite analysis[61] is highly interesting. Moreover, such an analytical system would make meaningful studies in chemical ecology feasible[1] which may lead to a combination of biochemical markers and/or genetic markers encoding enzymes involved in the biosynthetic pathways.

**5.2 Adaptation and Defense Mechanism:** Seagrasses grow in a wide range of salinity of 10 psu in the estuarine to higher salinities of 37 psu in the coastal environment. They grow from intertidal regions to a depth of above 60 m where minimal light exposure is found. Unless these organisms undergo certain biochemical adaptations they cannot withstand the competitive environment with different predators and organisms that are dependent on them. The concentration of polyunsaturated fatty acids was found to be lowest in *T. hemprichii* collected from warmer waters of Northern Queensland when compared to other seagrass species like *Zostera muelleri* and *Heterozostera tasmanica*[50] indicating that the seagrass adapt to temperature with a well regulated acetyl-coA pathway. The lipid and fatty acid composition of the seagrass *Z. marina* paves the way for the molecular thermoadaptation[56]. As stated earlier, their position of fatty acids on temperature scale may determine the capacity of poikilotherms to survive at the acute elevation of environmental temperature[62,63].

The phenolic compounds play an important role in the protection of seagrasses against competitors, predators and pathogens. Soluble phenolic acids shown to be abundant in a variety of seagrasses[14,41] and seagrass extracts are rich in soluble phenols which have been found to inhibit the growth of bacteria, fungi and algae[64-72]. The microbial growth and amphipod on the leaves of *Z. marina* was controlled by certain water soluble compounds produced by the host[73]. The chemical defense of the seagrass against pathogenic marine microbes is mainly exhibited by the flavones sulfates[35]. It was later confirmed that the seagrass *P. oceanica* in interaction with *C. taxifolia* accelerates its production of phenolic compounds so as to limit invasion of the beds[18]. Flavonoids are also found to be the most abundant secondary metabolites in seagrasses showing significant activities such as the feeding deterrence, antibacterial and antilarval activities[44].

**5.3 Biological Markers:** A wide profiling of seagrass secondary metabolites will help us to understand the food chain structure and biogeochemical characteristics of the seagrass communities. Several lipid components of the seagrass *P. oceanica* and *H. tasmanica* are suggested as potential markers for monitoring seagrass contribution to both food webs and marine sediments[74]. The absence of sulfated flavones and non-sulfated flavones are identified as marker to differentiate *Heterozostera* spp. from *Zostera* spp.[75] as the former contains other phenols than *Zostera* spp. The detection of lignin in seagrasses has also an implication for marine ecological studies where lignin is used as a land-derived marker[17] but the use of lignin as the marker for terrestrial origin of organic carbon in biogeochemical studies is not valued. Interestingly, the lipid n-alkane-2-ones are used as potential biomarkers to trace out the seagrass-derived organic matter[76]. The presence of long chain fatty acids (C22–C34) was identified as the marker to differentiate the *P. oceanica* residues from algal constituents. The lipid composition of seagrasses can be used widely for assessing the existing inter-relationship in the marine food web[54].

## 6 Outlook and Future Research

As evident from the above mentioned literature, a lot of basic research in the secondary metabolites of seagrasses still needs to be conducted for different species. In this context the search for additional sulfated phenols should be one that focuses on future research as such compounds were already detected in the seagrasses[36,38]. Moreover, the seacow (*Dugong dugon*) feed mostly on small, delicate seagrasses, especially *Halophila* and *Halodule*, and dig up whole seagrass plants including the roots[77]. Diet selection is correlated with the chemical and structural composition of seagrass[78] by *Dugong* which is mainly restricted to these small leaved seagrasses. Though the knowledge of secondary metabolites of these species is scarce when compared to *Posidonia* spp., it becomes necessary to explore the bioactive and nutrional value of all the seagrasses so as to recommend these values for biological and pharmaceutical application. It is well known that plants which possess anti-oxidative and pharmacological properties are related to the presence of phenolic compounds, especially phenolic acids and flavonoids[79]. Consequently, the plant might be a source of compounds to be investigated for anti-HIV and immunostimulant (caffeic acid, chicoric acid), antitumor (cinnamic acid, ferulic acid), antioxidant, and antibacterial activities[80]. All these facts suggest that seagrasses are a potential alternate source for novel biomedical metabolites. Also, the phenolic compounds gain attention as food preservatives, especially in the prevention of lipid peroxidation that causes food spoilage[19].

Presence, composition and quantity of phenolic compounds vary from species to species, in different parts of the same species, and in different age group of the same parts. The impact of environmental factors or cultivation conditions on the content of secondary compounds is immense. Different processing and instrumentation methods also lead to non-reproducible and non-comparable results among different laboratories. All these facts make the determination of phenolic compounds in seagrasses a challenging task. But one should consider the notion that better extraction procedures and purification techniques, coupled with advanced spectroscopic methods together, will pave the way to discover new and novel secondary metabolites of seagrass. There is only limited information regarding secondary metabolites for several seagrass species and no information is available for many species of the world. This division between limited information and no information makes it difficult to understand the pathways leading to the synthesis of secondary metabolites, their enzymes and regulation by external stimuli or their roles in plant defense mechanism[8]. To achieve this comparative phytochemical investigation both aimed at chemosystematic data for comparing tropical and temperate seagrass species is needed so as to answer questions in chemical ecology.

## 7 Concluding Remarks

A wide variation in the secondary metabolite composition of the marine angiosperms is observed within and between genera. The regulatory system for different pathways encoding for the secondary compounds should be explored at the gene level so as to understand the adaptive characters of the seagrasses. The secondary compounds like polyphenols, flavonoids and fatty acids are the key factors that are involved in the adaptation to changing biotic and abiotic environments and also mainly for the defense mechanism. The present review reveals that further profiling of secondary metabolites should be extensively progressed in order to obtain a deeper knowledge on their specific roles. Clearly such a wide array of secondary metabolites and their derivatives involves regulation by different genes through sophisticated mechanisms within the cell, which can be disclosed by the linkage of plant physiology, plant biochemistry and molecular biological aspects. The future of these secondary metabolites of seagrasses will likely result in surprising and unexpected advances in the characterization of new structures for useful development by mankind.

## Acknowledgments

We thank the Director Centre of Advanced Study in Marine Biology, Faculty of Marine Sciences, and the authorities of the Annamalai University for their support. We thank Chemspider database for chemical structures.

**Open Access** This article is distributed under the terms of the Creative Commons Attribution License which permits any use, distribution, and reproduction in any medium, provided the original author(s) and source are credited.
